# Environmental pollution impact on the severity of some rheumatic diseases: a comparative analytical study on inflammatory and non-inflammatory samples

**DOI:** 10.1186/s41927-024-00420-8

**Published:** 2024-10-08

**Authors:** Adel Elbeialy, Soaad El Sawy, Hala Elzomor, Rana Haddad

**Affiliations:** Rheumatology and Rehabilitation, Faculty of Medicine for Girls, Al Azhar University, Cairo, Egypt

**Keywords:** Heavy metals, Rheumatoid arthritis, Fibromyalgia, Vitamin D, Parathyroid hormone

## Abstract

**Objective:**

Environmental pollution of heavy metals is increasingly a problem and has become of great concern due to the adverse effects it causes worldwide. Heavy metal exposure has been implicated in health problems, including fibromyalgia and rheumatoid arthritis. We aim to evaluate the rule of chronic heavy metals toxicity on the induction of vitamin D3 (VD) deficiency and parathyroid hormone (PTH) disturbances in an inflammatory disease like rheumatoid arthritis (RA) and non-inflammatory disease like fibromyalgia syndrome (FMS).

**Methods:**

This comparative analytical study was conducted on sixty adults (age ≥ 18 years). Participants were divided into three groups. Group I: twenty patients diagnosed with RA according to the specific ACR/EULAR criteria for RA. Group II: twenty patients diagnosed with FMS according to the specific 2010 (ACR) criteria for FMS. Group III: twenty healthy adults. All patients and controls were subjected to routine laboratory tests as well as the measurement of PTH, VD and estimation of serum levels of lead, cadmium, and chromium.

**Results:**

VD was significantly inversely correlated to PTH, lead, cadmium, chromium, and activity scores in the RA and FMS groups. Lead, Cadmium and Chromium had a significant independent risk on the VD level in RA patients, while lead had a significant independent risk on the VD level in FMS patients.

**Conclusion:**

Heavy metals may affect VD synthesis, leading to hypovitaminosis D and secondary hyperparathyroidism in RA and FMS patients. Heavy metals play a key role in the pathogenesis of RA, FMS, and their disease activity.

## Key messages

1. Environmental pollution with heavy metals, can impact health conditions.

2. Rheumatic diseases including inflammatory ones like rheumatoid arthritis and non-inflammatory ones like fibromyalgia are mostly affected by chronic heavy metal intoxication.

3. Chronic toxicity with lead and cadmium has an impact on the pathogenesis of vitamin D3 deficiency and its autoimmune sequelae.

4. Chronic vitamin D3 deficiency and secondary hyperparathyroidism may have an important role in the pathogenesis of rheumatoid arthritis and fibromyalgia.

## Introduction

Rheumatoid arthritis (RA) is a chronic inflammatory autoimmune disease affecting the joints. It is characterized by a progressive symmetric inflammation of affected joints resulting in cartilage destruction, bone erosion, and disability. While initially only a few joints are affected, in later stages many joints are affected [1].

Fibromyalgia syndrome (FMS) is a chronic, non-inflammatory potentially disabling condition defined by core symptoms of widespread pain, fatigue, sleep disturbance and cognitive dysfunction [2].

Vitamin D (VD) is a fat-soluble vitamin well known for its essential role in bone health. It is converted in the body into several biologically active metabolites and interacts with the genome to produce both calcemic and nonracemic effects [3].

The function of VD in the homeostasis of calcium and phosphate plays an essential role in musculoskeletal health as its deficiency could lead to osteoporosis, osteomalacia, decreased bone mineral density, and increased risk of fragility fractures [4].

Vitamin D Deficiency (VDD) can be involved in the pathogenesis and/or progression of chronic illnesses, such as many common cancers (colon, breast, and prostate) autoimmune diseases, hypertension, cardiovascular diseases, and neurological disorders [5].

Human exposure to Heavy or toxic non-essential metals occurs from environmental pollution arising from the highest levels of these metals or due to their industrial use; considered to be harmful to humans and have become a major cause of illness, ageing and even genetic defects. Pollution by heavy metals occurs not only in anthropogenic areas but also in the distance from the sources of pollution [6].

A characteristic feature of heavy metals such as beryllium, mercury, lead, cadmium, aluminium, antimony, bismuth, barium, uranium and others, that increases danger is due to their accumulation and very slow excretion [7].

Chronic exposure to heavy metals, especially lead (Pb) and cadmium (Cd) affects the immune system. Heavy metals also decrease the number of existing B and T cells. The immune system attacks its self-molecules due to heavy metal exposure, which can lead to RA and other joint diseases [8].

Chronic metal poisoning usually presents with symptoms affecting multiple systems but is associated with three main types of symptoms: gastrointestinal, neuromuscular, and neurological. Signs include loss of short-term memory, depression, loss of coordination, numbness and tingling in the extremities, Fatigue, problems with sleep, headaches, stupor, and slurred speech, Many key symptoms of fibromyalgia and chronic fatigue resemble classic signs of heavy metal toxicity, including fatigue, neuromuscular pain, depression/anxiety, and sleep disturbances that may be shared by FMS symptoms [9].

Metals also can negatively affect steroid hormones, particularly VD and it has been demonstrated that heavy metals, especially cadmium (Cd) and lead (Pb) are capable of interfering with hormonal systems including the VD endocrine system [10].

We carried out this work to evaluate the rule of chronic heavy metals toxicity on induction of vitamin D3 deficiency and parathyroid hormone disturbances in fibromyalgia and rheumatoid arthritis patients and its correlation to their disease activity. This may add a new concept to the pathogenesis and treatment of these diseases.

## Patients and methods

This comparative analytical study was conducted on sixty adult participants (age ≥ 18 years) divided into three groups. Group I: twenty patients diagnosed with Rheumatoid Arthritis according to the 2010 American College of Rheumatology (ACR/EULAR) criteria [11]. Group II: twenty patients diagnosed with fibromyalgia syndrome (FMS) according to the 2010 American College of Rheumatology (ACR) criteria [12]. Group III: twenty control healthy adults. All participants were recruited from the Rheumatology and Rehabilitation Department of Al-Zahraa University Hospital, Al-Azhar University, Cairo, Egypt, during the period from November 2021 to September 2023. Informed written consent was taken from the patients after a detailed explanation of the study. This study and its protocol have been conducted according to regulations and approval of the Ethics Committee of the Faculty of Medicine for Girls, Al-Azhar University, Nasr City, Cairo, Egypt, Registered at Central Administration of Research and Development; Egyptian Ministry of Health: Reg No. RHBIRB2018122001 (approval date, May 2020, No. 202005236). The study was conducted following the principles of the Declaration of Helsinki.

Patients on calcium or vitamin D3 treatment, liver or kidney disease, current use of biologic therapy and other autoimmune diseases were excluded from the study.

All Patients were subjected to full history taking, Full clinical examination including general and musculoskeletal examination and full routine laboratory investigations (complete blood count, liver and kidney function tests, erythrocytes sedimentation rate, C-reactive proteins, rheumatoid factor, serum uric acid, random blood sugar, hepatitis-B virus and hepatitis-C virus, total and ionized calcium, parathyroid hormone, vitamin D3 and anti-CCP by ELISA technique).

###  Statistical analysis

Analysis of data was done by IBM computer using SPSS (statistical program for social science). Description of quantitative variables as mean, SD and range. Description of qualitative variables as number and percentage. Student t-test was done to compare between parametric data. Chi-square test to study the association between qualitative variables. Correlation analysis (using Pearson’s methods) to assess the strength of association between two quantitative variables the correlation coefficient denoted symbolically “r” defines the strength and direction of the linear relationship between two variables. Analysis of variance [ANOVA] tests. ANOVA test to study comparison among different times in the same group in quantitative data. Multivariate analysis (MVA) is based on the statistical principle of multivariate statistics, which involves observation and analysis of more than one statistical outcome variable at a time. ROC-curve to study the Sensitivity, Specificity, PPV, NPV and Accuracy.

## Results

Patients’ ages ranged from 38 to 53 years in the RA group with a mean of 47.8 ± 5.8 years, from 35 to 52 years in the FMS group with a mean of 45.5 ± 7.2 years, and from 36 to 51 years in the control group with a mean of 44.25 ± 5.16 years. The females were more in all groups: 19 (95%) in the RA and FMS groups and 20 (100%) in the control group. Regarding disease duration, in the RA group, it ranged from 1 to 7 years with a mean of 4,55 ± 2.50 years and in the FMS group ranged from 2 to 6 years with a mean of 4.1 ± 1.55 years (Table 1).

**Table 1:** Comparison between Groups as regards age, sex and disease duration

		Groups						*P*-value
		RA Group		FMS Group		Control Group		
**Age/years**	**Range**	38 - 53		35 - 52		36 - 51		0.190
	**Mean ± SD**^a^	47.8 ± 5.84		45.50 ± 7.273		44.250 ± 5.169		
**Sex**	**Male**	1	5%	1	5%	0	0%	0.596
	**Female**	19	95%	19	95%	20	100%	
**Duration/years**	**Range**	1 - 7		2 - 6		- - -		0.498
	**Mean ± SD**	4.550 ± 2.502		4.100 ± 1.553		- ± -		

^a^SD Standard deviation

As regards VIT D level in all groups, we found that there was a statistically significant decrease of VIT D in the rheumatoid group (21.845 ± 4.015 ng/ml) and the fibromyalgia group (23.932 ± 6.353 ng/ml) than in the control group (27.990 ± 5.303 ng/ml) where (*P* = 0.002) and (*P* < 0.001) respectively. There was no statistically significant difference between the rheumatoid and fibromyalgia groups (*P* = 0.433) (Table 2).

As regards PTH, There was a statistically significant increase of PTH in the RA group (77.030 ± 27.493 pg/ml) than in the FMS group (50.470 ± 12.503 pg/ml) (*P* < 0.001) and the control group (49.350 ± 8.028 pg/ml) (*P* < 0.001), but there was no statistically significant difference between the FMS group and the control group ( *P* = 0.979) (Table 2).

**Table 2:** Comparison between VIT D and PTH in study groups.

		Groups			ANOVA		TUKEY’S Test		
		RA Group	FMS Group	Control Group	F	*P*-value	I&II	I&III	II&III
**VIT D ng/ml**	**Range**	15.8–27.7	17–32	21.9–35.5	6.925	0.002*	0.433	0.002*	0.049*
	**Mean ± SD**	21.84 ± 4.01	23.93 ± 6.35	27.99 ± 5.30					
**PTH pg/ml**	**Range**	52–128	31.3–64.9	40–59	15.081	< 0.001*	< 0.001*	< 0.001*	0.979
	**Mean ± SD**	77.03 ± 27.49	50.47 ± 12.50	49.35 ± 8.02					

*VIT D* Vitamin D, *PTH* Parathyroid hormone

*Significant

The heavy metals (lead, cadmium, aluminium and chromium) differ significantly in the studied groups. We found that there was a statistically significant increase of Lead in the RA group than in the control group with *P* < 0.001; and a statistically significant increase in the FMS group than in the control group with *P* < 0.001 (Table 3).

In addition, there was a statistically significant increase of cadmium in the RA group than in the FMS group and control group with *P* = 0.009 and *P* < 0.001 respectively; and a statistically significant increase in the FMS group than in the control group with *P* < 0.001 (Table 3).

Chromium in the RA group showed a statistically significant increase than in the control group with *P* < 0.001; and a statistically significant increase in the FMS group than in the control group with *P* = 0.002 (Table 3).

**Table 3:** Comparison between heavy metals in studied groups

		Groups			ANOVA		TUKEY’S Test		
		RA Group	FMS Group	Control Group	F	*P*-value	I&II	I&III	II&III
**Lead ug/dl**	**Range**	8.6–11	8.2–11	7.3–7.5	65.187	< 0.001*	0.750	< 0.001*	< 0.001*
	**Mean ± SD**	9.66 ± 0.76	9.50 ± 0.88	7.47 ± 0.06					
**Aluminium ug/dl**	**Range**	3.7–7.4	2.8–8.4	4.6–4.8	0.256	0.775	0.977	0.767	0.875
	**Mean ± SD**	4.99 ± 1.06	4.90 ± 2.01	4.70 ± 0.046					
**Cadmium ug/dl**	**Range**	0.04–0.06	0.01–0.06	0.02–0.03	27.794	< 0.001*	0.009*	< 0.001*	< 0.001*
	**Mean ± SD**	0.055 ± 0.008	0.044 ± 0.018	0.028 ± 0.004					
**Chromium ug/dl**	**Range**	0.4–0.8	0.3–0.7	0.3–0.4	10.044	< 0.001*	0.918	< 0.001*	0.002*
	**Mean ± SD**	0.53 ± 0.13	0.52 ± 0.15	0.38 ± 0.04					

*Significant

There was a statistically significant inverse correlation between VIT D and all of PTH, Lead, Cadmium, Chromium, DAS 28, tender joints, swollen joints and patient global assessment (*p* < 0.001). On the other hand, there was a statistically significant direct correlation between PTH and all of Lead, Cadmium, Chromium, DAS 28, tender joints, swollen joints and patient global assessment (*p* < 0.001). In addition, there was a statistically significant direct correlation between heavy metals (Lead, Cadmium and Chromium) and DAS 28, tender joints, swollen joints and patient global assessment (*p* < 0.001) (Table 4).

**Table 4:** Correlation analysis between heavy metals and different parameters in the RA group

Correlations												
Group I	VIT D		PTH		LEAD		Aluminum		Cadmium		Chromium	
	*r*	*P*-value	*r*	*P*-value	*r*	*P*-value	*r*	*P*-value	*r*	*P*-value	*r*	*P*-value
**PTH pg/ml**	-0.885	< 0.001*										
**Lead ug/dl**	-0.969	< 0.001*	0.911	< 0.001*								
**Aluminium ug/dl**	-0.251	0.285	0.281	0.230	0.336	0.148						
**Cadmium ug/dl**	-0.901	< 0.001*	0.671	0.001*	0.803	< 0.001*	0.038	0.872				
**Chromium ug/dl**	-0.925	< 0.001*	0.921	< 0.001*	0.952	< 0.001*	0.398	0.082	0.692	0.001*		
**DAS 28**	-0.908	< 0.001*	0.854	< 0.001*	0.888	< 0.001*	0.082	0.732	0.849	< 0.001*	0.807	< 0.001*
**Tender Joints**	-0.775	< 0.001*	0.875	< 0.001*	0.842	< 0.001*	0.245	0.298	0.569	0.009*	0.795	< 0.001*
**Swollen Joints**	-0.716	< 0.001*	0.709	< 0.001*	0.687	0.001*	0.171	0.472	0.541	0.014*	0.717	< 0.001*
**PGA**	-0.876	< 0.001*	0.930	< 0.001*	0.908	< 0.001*	0.277	0.237	0.673	0.001*	0.883	< 0.001*

*VIT D* Vitamin D, *PTH* Parathyroid hormone, *DAS 28* Disease activity score 28, *PGA *Patient global assessment

*Significant

There was a statistically significant inverse correlation between VIT D and all of PTH, Lead, Cadmium, Chromium, SSS, WPI and VAS (*p* < 0.001). On the other hand, there was a statistically significant direct correlation between PTH and all Lead, Cadmium, Chromium, SSS, WPI and VAS (*p* < 0.001). In addition, SSS, WPI and VAS showed a statistically highly significant direct correlation with Lead and Chromium (*p* < 0.001), and a significant direct correlation with Cadmium (*p* = 0.007, 0.003, 0.004 respectively) (Table 5).

**Table 5:** Correlation analysis between heavy metals and different parameters in the FMS group

Correlations												
Group II	VIT D		PTH		LEAD		Aluminum		Cadmium		Chromium	
	*r*	*P*-value	*r*	*P*-value	*r*	*P*-value	*r*	*P*-value	*r*	*P*-value	*r*	*P*-value
**PTH pg/ml**	-0.897	< 0.001*										
**Lead ug/dl**	-0.914	< 0.001*	0.895	< 0.001*								
**Aluminium ug/dl**	-0.039	0.869	0.112	0.638	0.091	0.703						
**Cadmium ug/dl**	-0.690	0.001*	0.794	< 0.001*	0.770	< 0.001*	0.231	0.326				
**Chromium ug/dl**	-0.914	< 0.001*	0.965	< 0.001*	0.893	< 0.001*	0.199	0.401	0.728	< 0.001*		
**SSS**	-0.834	< 0.001*	0.878	< 0.001*	0.856	< 0.001*	0.112	0.638	0.586	0.007*	0.922	< 0.001*
**WPI**	-0.841	< 0.001*	0.915	< 0.001*	0.860	< 0.001*	0.097	0.686	0.623	0.003*	0.937	< 0.001*
**VAS**	-0.834	< 0.001*	0.881	< 0.001*	0.873	< 0.001*	0.048	0.840	0.619	0.004*	0.901	< 0.001*

*VIT D* Vitamin D, *PTH* Parathyroid Hormone, *VAS* Visual analogue scale, *SSS* Symptom severity score, *WPI W*idespread pain index

*Significant

## Discussion

The main aim of this study is to evaluate the rule of chronic heavy metals toxicity on the induction of vitamin D3 deficiency and hence parathyroid hormone disturbances in chronic inflammatory autoimmune diseases such as rheumatoid arthritis and non-inflammatory diseases like fibromyalgia and its correlation to disease activity. This may add a new concept to the pathogenesis and treatment of these diseases.

In our study, there was a statistically significant decrease of VIT D in the rheumatoid arthritis group than control, which was consistent with Abozaid et al. 2023 who found that the vitamin D levels in people with RA are statistically different from healthy control [13]. Also in the study done by Ismail 2022, the results reflect a remarkable decrease in the serum level of vitamin D of the Rheumatoid arthritis group in comparison to the control group [14]. Studies conducted by Kareem et al. 2015 and Yagiz et al. 2015 found remarkably lower Vitamin D concentrations in patients with RA as compared to the control population, thus supporting the potential role of (1,25-(OH)2D3) in the progression, development, activity and treatment of RA [15, 16].

As regards heavy metals in RA patients we found that there was a statistically significant increase of Lead, Cadmium and Chromium serum levels in RA patients than in healthy control with (*P* < 0.001) and no statistically significant difference in aluminium serum levels between the two groups (*P* = 0.775). Hashmi and Shah 2012 reported that lead, cadmium and chromium were statistically significantly higher in rheumatoid arthritis patients than in healthy control which agreed with our results [17]. Our results were supported by Afridi et al. 2015 who found a significant increase in lead and Cadmium in RA patients than in healthy control [18]. Also, another study by Irfan et al. 2017 found that Serum levels for lead, cadmium, and chromium were statistically significantly higher in RA patients than those in the healthy control (*P* < 0.01) [19]. In addition, our results were near to those of Joo et al. 2019 who found that serum Cd levels were increased in patients with RA compared with the control group [20].

In our study, there was no significant difference between RF positive and negative RA patients regarding heavy metals serum levels. Our results were supported by Yang et al.2016 who found no significant difference between RF positive and negative cases and lead, cadmium and chromium serum levels [21]. In addition, Arshad et al. 2023 found no significant difference between RF positive and negative cases and lead serum level [22].

In the present study, in the rheumatoid arthritis group, there was a statistically significant inverse correlation between VIT D and PTH, our study was supported by Elbeialy et al. 2021 who noticed a statistically significant inverse correlation between VIT D and PTH in rheumatoid arthritis patients [23].

In the current study, there was a statistically significant inverse correlation between VIT D and DAS 28 in the rheumatoid arthritis group. This outcome is similar to Soltani Bajestani et al., 2023 who studied the relationship Between Serum Vitamin D Level and Disease Severity in Rheumatoid Arthritis [24]. Another study by Attar 2012 on 100 RA patients and 100 controls, not on (1,25-(OH)2D3) supplements, noticed that patients with a high rate of RA activity had reduced (1,25-(OH)2D3) concentrations [25]. Haque and Bartlett 2010 and Rossini et al.2010 reported an inverse correlation between vitamin D levels and disease activity in RA [26, 27]. Also, Braun-Moscovici et al. 2011; Baker et al. 2012 did not state a relation between deficiency of vitamin D and disease activity in RA [28–30].

Our study showed that there was a statistically significant negative correlation between VIT D and PTH, Lead, Cadmium and Chromium in RA and FMS patients (*p* < 0.001). These results can be explained as Vitamin D increases the absorption of Cd and Pb which interfere with the renal activation of VTD and promotes lead toxicity [31].

Our study found a positive correlation between lead, Cadmium and chromium with disease activity (DAS28) in RA. These results align with Albabwaty et al. 2020 who found a positive correlation between lead, Cadmium and chromium with disease activity (DAS28) in RA [32].

This study showed a statistically significant decrease of VIT D in the fibromyalgia group than in the control group. Our study was supported by Akar et al. 2020 who found that patients with low VIT D levels had a higher frequency of fibromyalgia than those with normal VIT D levels [33]. Contrary to our study de Rezende Pena et al. 2010 found no statistically significant difference between the FM and control groups concerning mean serum concentration of 25-OHD and no correlation between vitamin D level and pain intensity [34]. Bellato et al. 2012; Okumus et al. 2013 found that Vitamin D3 deficiency and HPT are established to be associated with Fibromyalgia syndrome [35, 36].

In the current study, there was a statistically significant negative correlation between VIT D and PTH in the fibromyalgia group. That was similar to Elbeialy et al. 2021 who stated that the secondary hyperparathyroidism found in their patients is a sequence of chronic vitamin D3 deficiency or insufficiency which is not associated with renal problems, malignancies, or other precipitating factors. They attributed this chronic vitamin D3 deficiency or insufficiency to probable long-term exposure to pollutants, such as the heavy metals cadmium and lead, present in some mineral waters, fizzy waters, fried snacks, and tobacco smoke [37].

In addition, in our study, there was a statistically significant negative correlation between VIT D and VAS in the fibromyalgia group and that was the same way with Akar et al. 2020 who stated that patients with low vitamin D3 levels had higher pain levels [31]. Plotnikoff and Quigley 2003 reported that 89% of patients with chronic musculoskeletal pain had 25(OH)D3 deficiency [38].

In the current study, there was a statistically significant direct correlation between PTH and VAS in the fibromyalgia group and that was the same with Armagan et al. 2008 who measured serum PTH, calcium, phosphorus and active vitamin D in fibromyalgia patients [39].

## Conclusion

Heavy metals are highly implicated in the worldwide problem of vitamin D3 deficiency, and therefore the pathogenesis of many inflammatory autoimmune diseases like rheumatoid arthritis and non-inflammatory diseases like fibromyalgia and have a significant correlation to their disease activity. Hence, the measurement of heavy metals provides a new strategy for more accurately directed treatments of these diseases and high-risk groups. Avoiding exposure to heavy metal pollution in food and drink and chelation of heavy metals may provide more prospects for life-long freedom from ailments and disabilities.

So, we recommend further multi-center studies on a larger population to confirm our findings. Heavy metal pollution must be considered as one of the new millennium’s big challenges that should be treated efficiently, to prevent many economically affecting health hazards.

## Data Availability

All data generated or analyzed during this study are included in this published article.
